# The Practical Medicine Series of Year Books

**Published:** 1904-04

**Authors:** 


The Practical Medicine Series of Year Books. Issued Monthly. Volume 3. The Eye, Ear, Nose and Throat. Edited by Casey Wood, M.D.; A. H. Andrews, M.D.; G. P. Head, M.D.

This Volume of Practical Medicine Series attains the usual standard shown by those issued in previous years. It is a most excellent resume of the work done in the special branches of the eye, ear, nose and throat. We consider this work of very marked value to the man who wishes to keep up with all the advances made in this department, and who is not able to read or even have access to all the medical journals which publish the articles which are so excellently abstracted in these little volumes. It gives the most important, and at the same time tells you where to find the original article should you wish to read it more *in extenso.* We can certainly commend this volume.

